# Incidental cerebral aneurysms detected by a computer-assisted detection system based on artificial intelligence: A case series

**DOI:** 10.1097/MD.0000000000021518

**Published:** 2020-10-23

**Authors:** Yuki Shimada, Tetsuya Tanimoto, Masataka Nishimori, Antoine Choppin, Arie Meir, Akihiko Ozaki, Asaka Higuchi, Makoto Kosaka, Yuki Shimahara, Naoyuki Kitamura

**Affiliations:** a MNES Inc, Hiroshima; b Minamisoma Municipal General Hospital, Minamisoma; c Department of Neurosurgery, Fukushima Medical University, Fukushima; d Medical Governance Research Institute, Tokyo; e Kasumi Clinic, Hiroshima; f LPixel Inc, Tokyo, Japan; g Google Inc, Mountain View, California, USA; h Department of Breast Surgery, Jyoban Hospital of Tokiwa Foundation, Fukushima, Japan.

## Abstract

**Rationale:**

Computer-assisted detection (CAD) systems based on artificial intelligence (AI) using convolutional neural network (CNN) have been successfully used for the diagnosis of unruptured cerebral aneurysms in experimental situations. However, it is yet unclear whether CAD systems can detect cerebral aneurysms effectively in real-life clinical situations. This paper describes the diagnostic efficacy of CAD systems for cerebral aneurysms and the types of cerebral aneurysms that they can detect.

**Patient Concerns:**

From March 7, 2017 to August 26, 2018 we performed brain magnetic resonance imaging (MRI) scans for 1623 subjects, to rule out intracranial diseases. We retrospectively reviewed the medical records including the history and images for each patient.

**Diagnoses, interventions and outcomes:**

Among them, we encountered 5 cases in whom the cerebral aneurysms had been overlooked in the first and second round of imaging, and were detected for the first time by CAD. All missed aneurysms were less than 2 mm in diameter. Of the 5 aneurysms, 2 were internal carotid artery (ICA) paraclinoid aneurysms, 2 were Internal carotid-posterior communicating artery (IC-PC) aneurysms and 1 was a distal middle cerebral artery (MCA) aneurysm.

**Lessons:**

Our CAD system can detect very small aneurysms masked by the surrounding arteries and difficult for radiologists to detect. In the future, CAD systems might pave the way to substitute the workload of diagnostic radiologists and reduce the cost of human labor.

## 1 Introduction

Rupture of a cerebral aneurysm is the leading cause of subarachnoid hemorrhage, which can result in permanent neurological sequelae and can often be fatal. The estimated prevalence of unruptured aneurysms is approximately 3.2%.^[1]^ Unruptured cerebral aneurysms are often detected incidentally during cerebral artery imaging to rule out intracranial diseases in patients with suggestive symptoms.

Although digital subtraction angiography (DSA) is the gold standard for the diagnosis of aneurysm, unenhanced magnetic resonance angiography (MRA) has a sensitivity of 96.7%.^[2]^ In Japan, with the largest number of magnetic resonance imaging (MRI) devices per population among the countries in the Organization for Economic Co-operation and Development countries in 2018, MRA is routinely used to screen unruptured aneurysms as a noninvasive modality for health check-ups.^[2]^ However, it is often difficult to detect unruptured aneurysms depending on a lesion's characteristics such as the size and location, and it poses an enormous burden on the diagnostic radiologists to scan through a large number of MRI and MRA images in daily clinical practice, and ensure that there are no oversights.

Recent studies show that computer-assisted detection (CAD) systems based on artificial intelligence (AI) using convolutional neural network (CNN) have been successfully used for the diagnosis of various diseases, including skin cancer, glioma, lymph node metastases, diabetic retinopathy and acute neurologic illness.^[3–5]^ The technique is also used in MRA to screen for unruptured cerebral aneurysms, and a previous report of CAD system using CNN showed a sensitivity of 92.4%.^[6,7]^ We also independently developed another CAD system for MRA to screen for unruptured cerebral aneurysms, which showed a sensitivity of 91.1% with 4.7 false positives per case in a retrospective study performed on 656 cases.^[8]^

Our CAD system has been deployed in our clinic to assist the radiologists in diagnosis, and we performed 1623 brain MRI scans for imaging interpretation between March 7 2017, and August 26 2018. Among them, we encountered 5 cases of unruptured aneurysms which had been overlooked by two diagnostic radiologists but were detected only by the CAD system. In this report, we elucidate the characteristics of those lesions, to understand the differences in image interpretation between the human eye and the CAD system based on AI using CNN.

## 2 Materials and methods

### 2.1 Selection of cases

Our clinic is located in Hiroshima, Japan, and specializes in radiographic imaging diagnoses. From March 7, 2017, to August 26, 2018, we performed brain MRI scans for 1623 subjects to rule out intracranial diseases. Among them, we encountered 5 cases whose cerebral aneurysms had been overlooked in the first and second round of imaging interpretation, and were detected for the first time by CAD, before finalizing the diagnoses. We retrospectively reviewed the medical records including the history and images for each patient. Written informed consent was obtained from each patient. The present report of case series was exempt from ethical review of the institutional review board in accordance with the ethical guideline of medical studies.

### 2.2 Imaging techniques

Non-contrast enhanced 3D time-of-flight (TOF) MRA images were obtained by two MR units (PHILIPS 1.5T Achieva Release 3.2.3.4) using the following parameters: field of view (FOV), 170 mm, matrix size; pixel spacing; section thickness, 1 mm; section interval, 0.5 mm; repetition time (TR), 20 ms; echo time (TE), 3.1 ms. Maximum intensity projection (MIP) images were constructed by the following steps: (1) MIP processing by “Advanced Viewing”; (2) MIP image making; (3) creating three types of rotational images after deleting the external carotid arteries, each consisting of 15 projections evenly spaced around a single rotational axis (foot-to-head, right-to-left, and front-to-back).

### 2.3 CAD software

The CAD for cerebral aneurysms used in this study was developed by LPixel Inc. The algorithm consists of the following steps: (1) volume reconstruction from the MRA images; (2) vessel segmentation using a threshold-based method; (3) key point extraction based on a principal curvature method^[9]^; (4) ranking of key points using a deep learning method (ResNet-18, a convolutional neural network^[10]^); (5) clustering of the key points with a score higher than 0.5. This CAD was operated on the imaging interpretation system of MNES Inc., LOOKREC, which is an application program on the Google Cloud Platform. All the radiographic images acquired by MNES Inc. were uploaded and interpreted on LOOKREC.^[11]^

### 2.4 Imaging interpretation procedure

In the first step, one of the diagnostic radiologists in our group interpreted the MRA images. Second, the last author (N.K.) interpreted them in reference to the first imaging interpretation. Finally, N.K. checked the output of the CAD system and finalized the diagnoses.

## 3 Results

**Figure 1:**
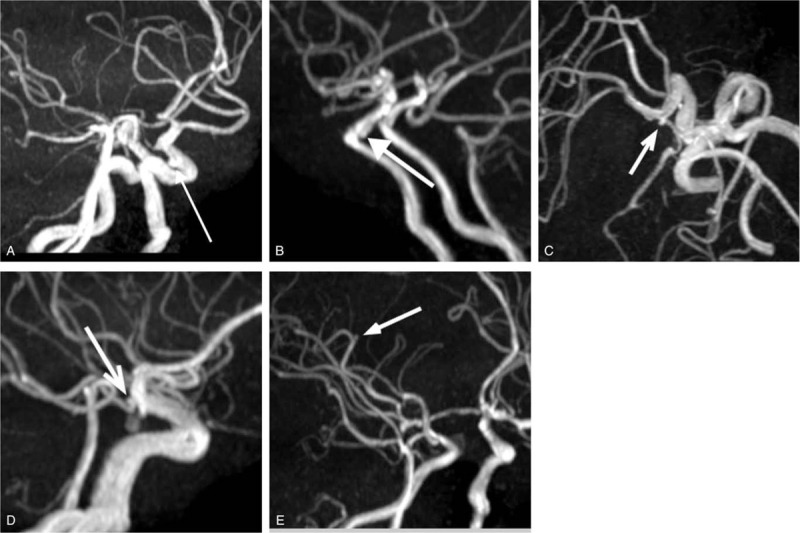
MIP images of 5 cases. A and B, The MIP images depict small IC-paraclinoid aneurysms (arrows, Cases 1 and 2). C and D, The MIP images depict small IC-PC aneurysms (arrows, Cases 3 and 4). E, The MIP image depicts a small right distal MCA aneurysm.

Table 1 summarizes the demographic and aneurysmal characteristics of the 5 patients. All the patients were females and visited our clinic due to some symptoms; 2 of them complained of headache, 2 complained of dizziness and 1 complained of numbness in the hands. The internal carotid artery (ICA) paraclinoid aneurysms were missed by the radiologists in Case 1 and Case 2 (Fig. 1A and B). The internal carotid-posterior communicating artery (IC-PC) aneurysms were missed in Case 3 and Case 4 (Fig. 1C and D). A distal middle cerebral artery (MCA) aneurysm was missed in Case 5. All missed aneurysms were less than 2 mm in diameter.

**Table 1:**
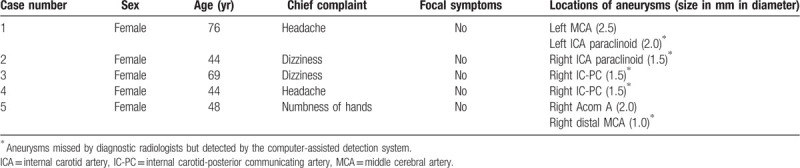
Characteristics of patients.

## 4 Discussion

We reported here, the details of small cerebral aneurysms that were overlooked by two diagnostic radiologists and detected only by CAD based on AI using CNN. These cases suggest that CAD for cerebral aneurysms can potentially reduce the incidence of missed cerebral aneurysms, because some anatomical characteristics and size of cerebral aneurysms might be difficult to detect by the human eyes, depending on a specialist's ability as well as fatigue from reading a large number of images for a prolonged period of time.

All the 5 cases of missed unruptured cerebral aneurysms were less than 2 mm in diameter. Two were IC-PC aneurysms, which had the fetal type of posterior communicating artery (PcomA) This aneurysm was detected only on a single rotational view. Two others were IC paraclinoid aneurysms that were located on the dorsal side of the ICA and detectable only on the lateral view. They had a wide neck and were seemingly a vasodilation of an artery. The fifth was a distal MCA aneurysm, located peripherally and could be difficult to detect. As such, the size of an ICA aneurysm can be relatively smaller than the diameter of the ICA, and such aneurysms might be obscured on most MIP images and are difficult to detect by the human eye. Our CAD system might be especially useful for detecting such lesions.

To prevent missing very small aneurysms less than 2 mm in size and likely to be obscured by the surrounding arteries, it would be effective to obtain MIP images with various views. However, increasing the number of images than the current standard is challenging, considering the additional workload and time in routine clinical practice. On the contrary, the CAD system can process a larger number of MRI images in less than one minute per subject, with stable processing ability and without any fatigue, which is difficult for human beings. Thus, to deploy the CAD system in conjunction with the screening by diagnostic radiologists would effectively reduce the incidence of missing a very small aneurysm as shown in our case series.

This study is the first study to illustrate the characteristics of cerebral aneurysms which were detected only by CAD based on AI using CNN. However, there are several limitations. First, all of them are very small aneurysms. Arguably, aneurysms less than 2 mm in diameter have a relatively low risk of rupture. Therefore, cerebral aneurysms detected only by CAD might not have big clinical importance. However, if they grow over time, the risk of aneurysm rupture per patient-year can be as high as 2.4%, which is twelve times higher than the risk of rupture in aneurysms that do not increase in size.^[12]^ A long-term follow-up of very small cerebral aneurysms is necessary to check on their rapid enlargement. In addition, it is important to preemptively offer intervention for risk factors of cerebral aneurysm rupture, such as hypertension and smoking.^[13]^ Therefore, detecting such small aneurysms might be beneficial for some patients, although the cost-benefit analysis needs to be explored to completely justify this approach in the future. Second, these cerebral aneurysms detected only by CAD were not diagnosed by Digital subtraction angiography or computed tomography angiography. Due to the limited spatial resolution and artifacts such as motion, susceptibility, and flow of MRA, cerebral aneurysms which were detected only by CAD might be false-positive.

In a recent study by another group, their CAD systems using different algorithms compared to the one used by us, also showed a high sensitivity for detection of cerebral aneurysms; however, their dataset comprised of the population examined by one model of MRI apparatus in only one hospital, while their CAD system was validated by using another dataset of the same population which used as training data.^[6]^ In contrast, our CAD was developed with a 3D CNN algorithm from 3D TOF MRA raw data and validated by using another dataset completely different from training data, and not from the MIP data of various types of MRI apparatus. In addition, we demonstrated the usefulness of our CAD in a setting in which two diagnostic radiologists interpreted the same MRA, to prevent missing the diagnosis in referred symptomatic patients in routine clinical practice. In the future, it is necessary to compare the ability of different algorithms of each CAD system using the same dataset, which might pave the way to substitute the workload of diagnostic radiologists and reduce the cost of human labor.

## Acknowledgments

We offer special thanks to Dr Shigeaki Kato and Dr Tomoyoshi Oikawa for their useful and constructive opinions on this study. We also would like to thank tha staffs at Kasumi Clinic and MNES Inc.

## Author contributions

**Conceptualization:** Yuki Shimada, Tetsuya Tanimoto.

**Data curation:** Yuki Shimada, Tetsuya Tanimoto, Makoto Kosaka.

**Investigation:** Yuki Shimada, Tetsuya Tanimoto, Akihiko Ozaki.

**Methodology:** Yuki Shimada.

**Project administration:** Yuki Shimada, Tetsuya Tanimoto.

**Software:** Masataka Nishimori, Antoine Choppin, Yuki Shimahara, Naoyuki Kitamura.

**Supervision:** Tetsuya Tanimoto, Masataka Nishimori, Arie Meir, Yuki Shimahara, Naoyuki Kitamura.

**Writing – original draft:** Yuki Shimada.

**Writing – review & editing:** Tetsuya Tanimoto, Masataka Nishimori, Antoine Choppin, Arie Meir, Akihiko Ozaki, Asaka Higuchi, Makoto Kosaka, Yuki Shimahara, Naoyuki Kitamura.
